# Detection and classification of ChatGPT-generated content using deep transformer models

**DOI:** 10.3389/frai.2025.1458707

**Published:** 2025-04-04

**Authors:** Mahdi Maktabdar Oghaz, Lakshmi Babu Saheer, Kshipra Dhame, Gayathri Singaram

**Affiliations:** Faculty of Science and Engineering, Anglia Ruskin University, Cambridge, United Kingdom

**Keywords:** natural language processing, ChatGPT, large language models, Generative AI, transformers, BERT, text classification

## Abstract

**Introduction:**

The rapid advancement of AI, particularly artificial neural networks, has led to revolutionary breakthroughs and applications, such as text-generating tools and chatbots. However, this potent technology also introduces potential misuse and societal implications, including privacy violations, misinformation, and challenges to integrity and originality in academia. Several studies have attempted to distinguish and classify AI-generated textual content from human-authored work, but their performance remains questionable, particularly for AI models utilizing large language models like ChatGPT.

**Methods:**

To address this issue, we compiled a dataset consisting of both human-written and AI-generated (ChatGPT) content. This dataset was then used to train and evaluate a range of machine learning and deep learning models under various training conditions. We assessed the efficacy of different models in detecting and classifying AI-generated content, with a particular focus on transformer-based architectures.

**Results:**

Experimental results demonstrate that the proposed RoBERTa-based custom deep learning model achieved an F1-score of 0.992 and an accuracy of 0.991, followed by DistilBERT, which yielded an F1-score of 0.988 and an accuracy of 0.988. These results indicate exceptional performance in detecting and classifying AI-generated content.

**Discussion:**

Our findings establish a robust baseline for the detection and classification of AI-generated textual content. This work marks a significant step toward mitigating the potential misuse of AI-powered text generation tools by providing a reliable approach for distinguishing between human and AI-generated text. Future research could explore the generalizability of these models across different AI-generated content sources and address evolving challenges in AI text detection.

## 1 Introduction

The growth of Artificial Intelligence (AI) specifically Artificial Neural Networks (ANN) tools has been one of the most significant technological advancements of the past few decades. With the availability of massive amounts of data and the ever-increasing computing power, AI has become a vital tool in solving complex problems and making predictions across various industries. The advent of deep learning has revolutionized the field of artificial intelligence (Aggarwal et al., 2022). Deep learning has led to significant breakthroughs in areas such as computer vision, natural language processing, and speech recognition. It has enabled the development of new applications such as autonomous vehicles, virtual assistants, and medical diagnostics. Despite its many benefits, there are also potential adverse effects associated with this powerful tool (Helbing, 2019). AI applications can be misused in various ways. Violation of privacy through AI-powered surveillance and facial recognition technologies, use of AI to automate social media content moderation which can result in censorship and restrict freedom of speech, use of AI to create fake news or deep-fake videos, which could spread misinformation, shift the public narrative and defame individuals are just some examples of the adverse implications of Artificial Intelligence (Brundage et al., 2018; Javadi et al., 2020; Khisamova et al., 2019).

AI-powered text generative tools aka chatbots are one of the most important applications of deep learning. These tools rely on natural language processing and machine learning algorithms to understand and interpret user input and provide relevant responses in real-time. Previously chatbots could only answer specific short queries and hold small domain-restricted conversations. The current GPT/BERT transformer-based model chatbots can analyze long queries and generate lengthy responses in real-time, thanks to the inherent mechanisms of the transformer-based models. They can deal with various types of text content, including emails, recipes, poems, meeting summaries, essays, and even algorithms or code. Modern chatbots can analyse the context of a conversation, learn from past interactions, and continuously improve their responses, providing a more personalized and efficient experience for the users (Adamopoulou and Moussiades, 2020; Arsenijevic and Jovic, 2019). Chatbots can provide fast and convenient customer support, handle multiple inquiries at once, are available 24/7, and reduce business operational costs.

Despite the numerous benefits of these tools, they can be misused in various ways. AI-powered text-generative tools can be used for malicious purposes such as spreading false information, promoting scams and phishing attacks, and fabricating assessment solutions in academia. On top of that, there are significant concerns regarding the reliability and correctness of the contents that are sourced from these technologies (Kooli, 2023; Tlili et al., 2023). As more people rely on these tools for generating content, it hampers the creativity of individuals, the diversity of ideas, and the credibility of cyberspace. Excessive use of AI-powered text generation models in academic settings could negatively affect the growth and development of students. Although these tools offer an easy way to access information, they lack the credibility of peer-reviewed books and scientific articles authored by subject experts. Over-reliance on these tools leads to a lack of critical thinking skills and independent learning among learners. In addition, it makes it difficult to assess the quality and originality of the work submitted by the students and evaluate their comprehension, which often leads educators to resort to less accessible assessment methods such as timed examinations and presentations (Bozkurt et al., 2023; Tlili et al., 2023).

In this regard, this study aims to investigate the performance of various machine learning/deep learning models and text classification pipelines in detecting and classifying textual content generated using AI. Our specific focus in this study is on the textual content generated by OpenAI's ChatGPT, which is widely regarded as one of the most powerful and accessible tools in this field. To realize this goal, we collected and compiled a large dataset comprising both ChatGPT and human-generated textual content in the field of computer science and networks. We used this dataset to train several machine learning models including Multinomial Naive Bayes, Support Vector Machines (SVM), K Nearest Neighbor, Random Forest, and a number of state-of-the-art deep learning models including a baseline Bidirectional Long Short Term Memory (BiLSTM) model, DistilBERT (Sanh et al., 2019), RoBERTa (Liu et al., 2019), and a custom deep model and evaluate their performance in classification of AI-generated (ChatGPT) textual content under various experimental setup and hyperparameters.

The main contribution of this study is the collection and compilation of a large dataset consisting of textual content, which includes both ChatGPT-generated and human-generated question-answers in the field of computer science, networks, and security. This dataset has been made available publicly for research purposes (for more details please refer to Section 3). This study particularly focuses on the detection of AI-generated content in academia particularly in the field of computer science to safeguard academic integrity and detect plagiarism. Furthermore, this study contributes to establishing a baseline for the detection and classification of AI-generated textual content in academia using state-of-the-art machine learning and deep learning models. This study also offers a comparative analysis between the proposed classification models and Turnitin's AI plagiarism detector, one of the most widely recognized solutions in the field, thus making an additional contribution to the field. Exhaustive experiments are performed to identify the right set of features, processes, algorithms, and hyperparameters to optimize the proposed models.

The rest of the manuscript is structured as follows: Section 2 offers a closer look at the literature and investigates and scrutinizes various related studies; Section 3 outlines the proposed dataset structure, data collection, and compilation process; Section 4 explains the proposed method including different text mining pipelines, classification models, their hyper-parameters, and training regimes utilized in this research; Section 5 summarizes the results of the study and finally, Section 6 provides the concluding remarks.

## 2 Literature review

Textual content generation has gained substantial popularity in recent years, primarily due to advancements in AI and deep learning. Numerous commercial and open-source tools and technologies are now readily available to facilitate the process of generating plausible textual content. Despite the countless benefits of these tools, they come with potential adverse side effects and can be often misused. Therefore, it is crucial to study measures for the identification of AI-generated content and address ethical concerns to promote responsible and accountable practices in the field. This is especially important when it comes to academia where integrity and authenticity are crucial. Over the last decade, many studies attempted to differentiate and classify AI-generated textual content from original human-generated work. This section briefly outlines some of these attempts and addresses their possible shortcomings.

A study by Tien and Labbé (2018), attempts to detect computer/AI-generated sentences and short textual fragments using Grammatical Structure (parse tree) Similarity (GSS). This study particularly focuses on textual contents generated using Probabilistic Context Free Grammar (PCFG), Recurrent Neural Network (RNN), and Markov models. This research used multiple PCFG corpora paired with the Jaccard similarity index to evaluate the performance of the proposed model. This model is claimed to achieve an 80% positive detection rate and less than a 1% false detection rate. A similar study by Labbé et al. (2016) investigates the use of Markov Chain and PCFG approaches in the generation of computer/AI-generated scientific texts, with a focus on the SCIgen tool. They managed to successfully detect computer/AI-generated contents by leveraging three different factors; vocabulary richness, length and structure of sentences, and frequency of word distribution with a combination of Inter-textual Distance and Agglomerative Hierarchical Clustering algorithms. It was concluded that computer/AI-generated SCIgen fails to diversify the vocabulary depending on the situation, thereby making it easier for detection.

In another study, Lavoie and Krishnamoorthy (2010) attempted to detect academic papers generated using the SCIgen software. This study primarily focuses on keywords occurrences in different sections of the papers. First, it computes the title and abstract score which denotes the frequency of occurrence of keywords in these sections of the paper. Second, the word repetition score extracts the top *N* most common words and Finally, the references score denotes the occurrence of words in the citations mentioned in the paper. Despite a limited sample size of 200 papers, this study was claimed to be able to successfully detect computer-generated scientific papers using K-Nearest Neighbor Algorithm. On the downside, this model appears to be computationally expensive while taking only a limited number of features into account for the detection of computer-generated academic papers.

In a slightly different study, Nguyen-Son and Echizen (2018) proposed a novel model for predicting computer-generated text using noise and language fluency factors. This model was deployed on 1,000 human-written English messages along with 1,000 Google-translated (computer-generated) Spanish messages to extract the fluency and noise features. The fluency features were extracted by measuring *N*-grams and their frequency whereas, the noise features were retrieved by extracting candidate noises, spoken word noises, and unexpected noise words followed by calculating the minimum edit distance of unexpected noise words. Combining the two characteristics allowed the Sequential Minimal Optimisation (SMO) classifier to classify computer-generated text with up to 80.35% accuracy accurately. The detection of more complex and longer computer-generated content may not be possible as this study focuses on short text messages, which often contain slang and untranslated phrases.

In another study, Nguyen-Son et al. (2017) proposed a new method for recognizing computer-generated textual content using statistical analysis to address shortcomings in their earlier work. The frequency, complexity of phrases, and consistency of words within a document were taken into account during the feature extraction process. Nine distinct features were produced by these three feature extraction techniques, and they were then utilized to classify human-written textual content from computer-generated counterparts. Logistic Regression, Support Vector Machine (SVM), Sequential Minimal Optimisation (SMO), and Stochastic Gradient Descent (SGD) classifiers were investigated in this research. It was discovered that SVM with SGD achieved the highest accuracy of 89.0% on the ancient Complex phrases feature alone. After merging all the data, the model was able to reach an accuracy of 98%. Due to the widespread use of Neural Language Models in producing computer-generated text, it has become more challenging to distinguish computer-generated text from content written by humans which is necessary for many educational and creative industries. A study by Ifeoluwa Adelani et al. (2019) focused on the identification of fake product reviews in online e-commerce and retail outlets. The research employed the GPT-2 Neural Language Model (NLM) to create numerous high-quality reviews that aligned with a specific sentiment. The reviews were then screened for undesired sentiments using a BERT-based text classifier, which had an accuracy rate of 96%. Further, the study analyzed the reviews with Grover, GLTR and OpenAI GPT-2 model, but was unable to achieve satisfactory results. A similar study by Stiff and Johansson (2022) attempted to detect computer-generated disinformation in news articles and social media posts. This study evaluated a number of state-of-the-art Transformer-based detection algorithms at various configurations. Authors reported that the majority of these detectors are unable to generalize and detect computer-generated short social media posts and are vulnerable to basic adversarial attacks.

A number of other studies attempted to investigate how AI-generated textual content can negatively impact academia and how its adverse effects can be mitigated. A study by Abd-Elaal et al. (2019) explored the use of Artificial Intelligence for academic misconduct, plagiarism, falsification, and fabrication of academic articles. This study shows how Automatic Article Generator (AAG) tools use keywords, articles, or topics from users to generate documents through the gradual propagation of the related articles in the form of a knowledge tree. AAGs make use of advanced natural language processing and deep learning techniques to frame human-like sentences. This study indicates the use of AAG tools in academia imposes challenges for evaluators to understand the true potential of the students and researchers. Similarly, Jiffriya et al. (2021) attempted to investigate the use of AI in the context of plagiarism in academia. This study offers a survey on plagiarism detection tools and techniques for computer code and natural language. It classifies natural language into four major categories based on the mode of corporal, type of application, mode of service and language. They identified the majority of plagiarism detection tools are suffering from three major issues: firstly, the scope of detection, some tools compare documents that are only present in their own repository while others face issues with accessing password-protected databases on the Internet. Secondly, analyzing paraphrased text is a challenge as they use synonyms, rewording, and reordering. Finally, documents translated from one language to another are hard to be detected by plagiarism tools. This study suggests the need for more advanced tools for detecting plagiarism in academia as AI-powered computer-generated tools are getting more accessible to students.

Many researchers attempted to investigate methods used for identifying computer-generated textual content. A study by Beresneva (2016) offers a systematic review of computer-Generated text detection using machine learning techniques. Various methods like frequency analysis, linguistic feature analysis, phrase analysis, lexicographic feature analysis, and hidden style similarity have been investigated and compared in this study. The author suggested characteristics of computer-Generated textual content are the primary factor in finding the right detection model.

A study by Gruner and Naven (2005) offers a tool for plagiarism detection that mainly relies on Morton's Word Pattern Ratios (Morton, 1978). The functionality of this tool can be described in four sequential methods: Converting formatted text into processable plain text, Testing the texts pairwise for each of 62 Morton's Word Pattern rules, Comparing single test results and counting the matches, and If the matching threshold is reached, issue a warning. Promising findings in terms of stylometric detection accuracy have been reported. However unorthodox nature of this study, makes it difficult to compare it with other literature. A similar study by Lukashenko et al. (2007) discusses approaches to mitigate plagiarism and investigates tools for detecting plagiarism. This study indicates that the majority of the existing plagiarism detection approaches rely only on simple statistical and frequency-based techniques. This study indicates, while these tools perform well in finding similarities among documents, they are not yet able to detect computer-generated content.

A study by Arabi and Akbari (2022) proposed two methods to identify extrinsic plagiarism. To minimize the search space, this study used two stages of filtering at both document and sentence levels based on the Bag of Word (BoW) technique. The first method used a combination of pre-trained FastText words embedding and TF-IDF to detect similarities while the second method benefits WordNet ontology and weighting TF-IDF techniques. Experimental results on the PAN-PC-11 corpus show that the first method achieved 95.1% precision and the second method 93.8% precision which evidences the advantage of the word embedding method for automated plagiarism detection. A study by Khaled and Al-Tamimi (2021) looks at academic plagiarism and plagiarism detection approaches in a comparative study. A number of plagiarism detection tools such as MOSS, Turnitin, DupliCheck, and PlagScan have been investigated and compared. This study concludes that paraphrasing, repetitive research, secondary source, duplication, and verbatim are the most common types of academic plagiarism. This study also outlines some challenges in plagiarism detection including the absence of an accurate framework for AI plagiarism detection that can reveal text segments for both plagiarism detection.

There are several other studies (Khalil and Er, 2023; Apoorv et al., 2020; Eissen and Stein, 2006; Potthast et al., 2010) that concern the detection of AI-generated content for academic plagiarism detection purposes, however, there is still a need for further research and development in this area as AI technology continues to evolve and improve. Current methods of detecting AI-generated textual content may not be sufficient to keep up with the sophistication and diversity of AI-generated content. Additionally, as AI-generated content becomes more prevalent and accessible, it may become increasingly difficult to distinguish between human-generated and AI-generated content. Therefore, continued research and development in this area is crucial to address these challenges and ensure the integrity and reliability of information especially in an academic context.

## 3 Transformer-based architectures: BERT and GPT models

The superior performance of models like BERT and GPT is rooted in the architecture of the transformer model, which fundamentally relies on self-attention mechanisms and a feed-forward network architecture. Unlike recurrent neural networks (RNNs) and LSTMs, which process sequences in a stepwise fashion and are constrained by the vanishing gradient problem when handling long-range dependencies, transformers allow for complete parallelization across the input sequence, significantly increasing computational efficiency and scalability.

BERT is built upon a stack of identical transformer encoder layers, each of which consists of multi-head self-attention followed by a position-wise fully connected feed-forward network. The self-attention mechanism allows the model to compute the importance of each token relative to all other tokens in the input sequence, capturing both local and global dependencies. The “multi-head" aspect means that multiple attention distributions are learned in parallel, allowing the model to focus on different parts of the input simultaneously. This is crucial for understanding the bidirectional context, which is one of BERT's key innovations. BERT's architecture typically includes 12–24 encoder layers (or “transformer blocks"), with each layer having 12 or 16 attention heads, depending on the model size (BERT-base vs. BERT-large). The hidden size of each layer is 768 for BERT-base and 1,024 for BERT-large, with a corresponding number of parameters (110M for BERT-base and 340M for BERT-large). The pre-training of BERT involves tasks like Masked Language Modeling (MLM), where random words are masked, and the model is trained to predict them, and Next Sentence Prediction (NSP), which helps the model understand sentence-level relationships.

GPT, on the other hand, employs a decoder-only transformer architecture, optimized for autoregressive tasks. The model processes the input text in a unidirectional fashion, where each token attends only to the tokens preceding it in the sequence. GPT's transformer layers are similarly composed of multi-head self-attention and feed-forward networks, but unlike BERT, GPT does not include bidirectional context. This unidirectional nature allows GPT to excel in generative tasks by predicting the next word in a sequence based on the preceding words. GPT models like GPT-3 utilize up to 96 transformer layers, with a hidden size of 12,288 and over 175 billion parameters, enabling the model to generate human-like text across a wide range of tasks. The use of LayerNorm before each sub-layer and residual connections between layers ensures stable gradient propagation and prevents degradation as the model depth increases.

Both BERT and GPT architectures employ positional encodings to retain information about the order of tokens, which is crucial for tasks where word order impacts meaning. The transformer's reliance on self-attention allows these models to scale effectively with larger datasets and deeper architectures, which, combined with their pre-training on massive corpora and subsequent fine-tuning on specific downstream tasks, results in state-of-the-art performance in natural language understanding and generation.

## 4 Data collection process

**Figure 1:**
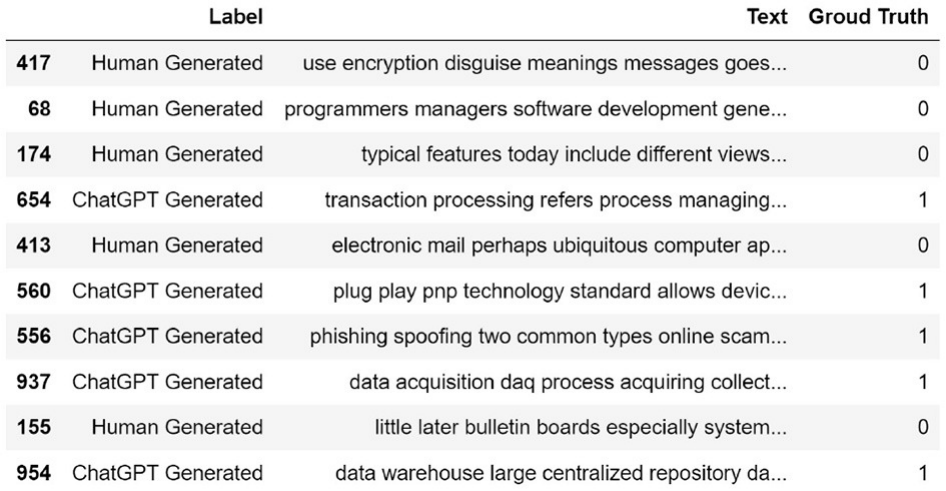
A random sample of the proposed dataset. ChatGPT-generated entries are labeled as 1 while Human-generated entries are labeled as 0.

**Figure 2:**
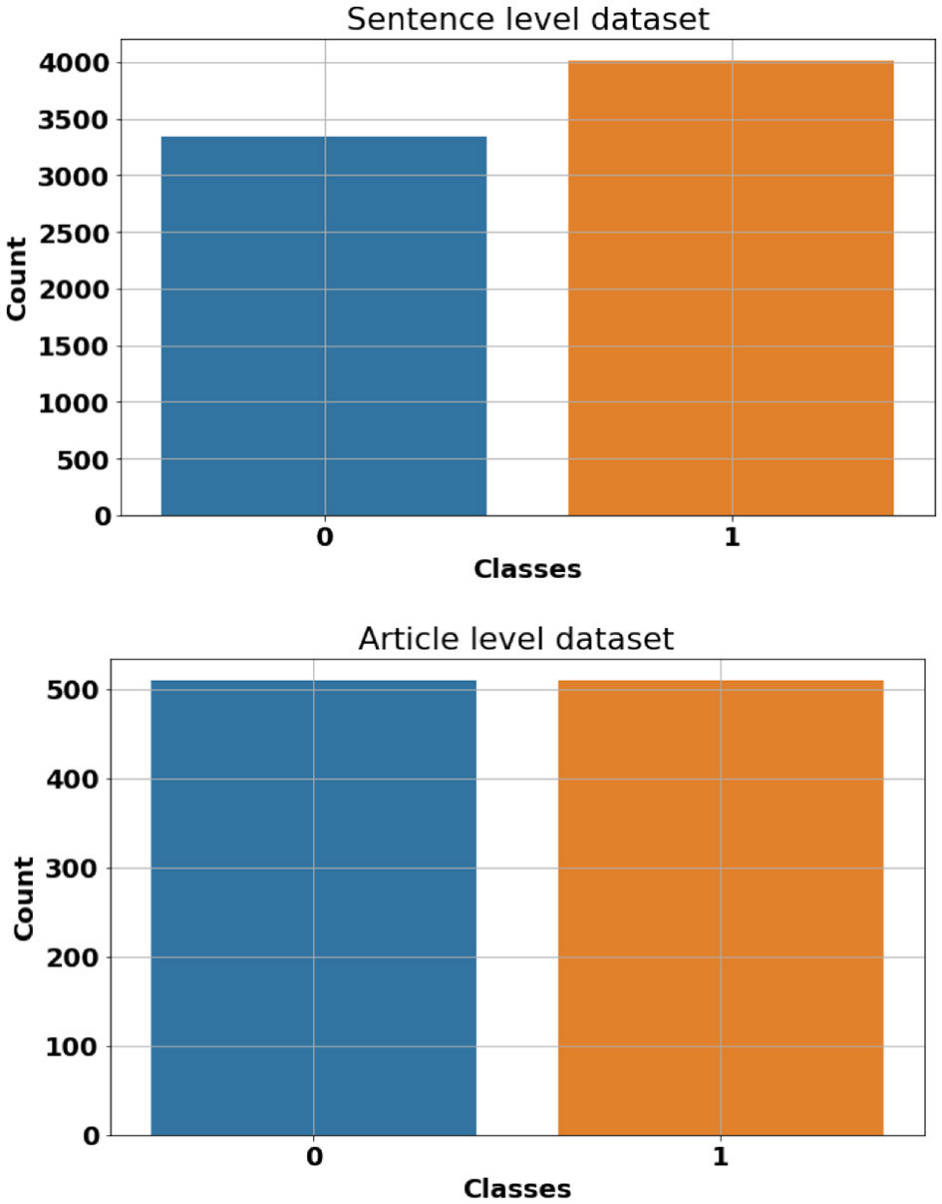
Class frequency count across both article-level (**bottom**) and sentence-level (**top**) datasets. Class 0 represents Human-generated contents while class 1 denotes AI-generated (ChatGPT) contents.

In order to carry out this research, we have compiled a dataset that consists of 509 descriptive question-answers about common terminology, concepts and definitions in the field of computer science, artificial intelligence, and cyber security. These questions were answered using both human-generated content and OpenAI's ChatGPT engine. Human-generated answers were collected from different computer science dictionaries and encyclopedias including “*Encyclopedia of Computer Science and Technology"* (Henderson, 2009) and “*Encyclopedia of Human-Computer Interaction”* (Ghaoui, 2005). AI-generated content in our dataset was produced by simply posting questions to OpenAI's ChatGPT and manually documenting the resulting responses. A rigorous data-cleaning process has been performed to remove unwanted Unicode characters, styling and formatting tags. Although the focus of this study is mainly on the classification of the AI-generated and Human-generated content, the question-answers nature of this dataset allows additional possibilities for our future research. To restructure our dataset for binary classification, we combined both AI-generated and Human-generated answers into a single column and assigned appropriate labels to each data point (Human-generated = 0 and AI-generated = 1). This creates our *article-level* dataset which consists of a total of 1,018 articles (answers), 509 AI-generated and 509 Human-generated. Additionally, we have divided each article (answer) into its sentences and labeled them accordingly. This is mainly to evaluate the performance of classification models and pipelines when it comes to shorter sentence-level data points. This constructs our *sentence-level* dataset which consists of a total of 7,344 entries (4,008 AI-generated and 3,336 Human-generated). Figure 1 shows a random sample of the proposed dataset. Also, Figure 2 shows class frequency count across both article-level and sentence-level datasets.

**Figure 3:**
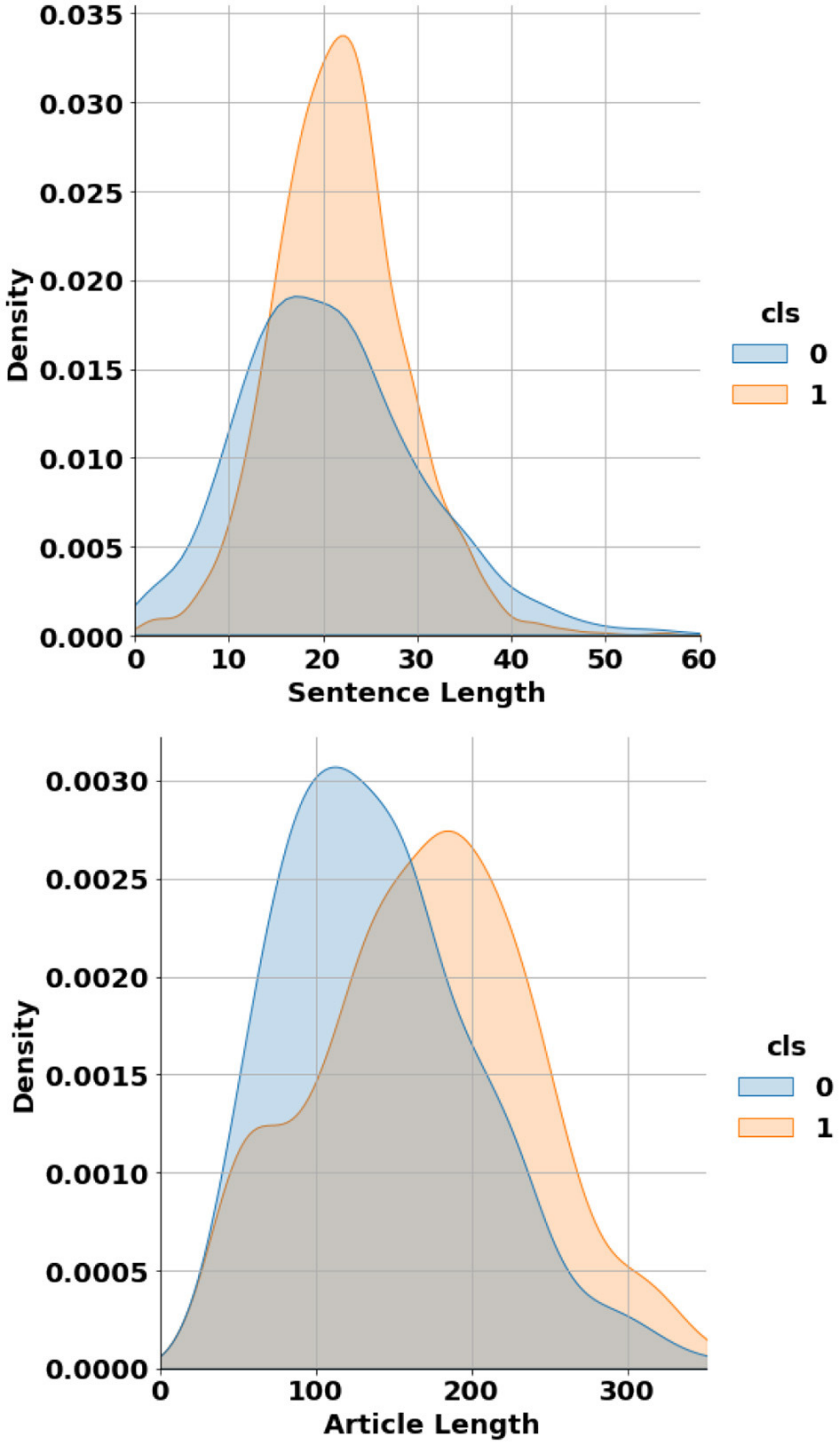
Probability distribution of data entries' length across both article-level (**bottom**) and sentence-level (**top**) datasets.

In terms of length, the article-level dataset contains individual articles ranging from 26 to 456 words, while the sentence-level dataset includes sentences varying from 1 to 171 words. Figure 3 shows the probability distribution of data points length across both article-level and sentence-level datasets. It can be seen that on average, AI-generated sentences and articles tend to be longer than their Human-generated counterparts. The proposed dataset has been made publicly accessible and can be downloaded from supplementary documents of this article.

## 5 Methodology

This study explores several NLP pipelines and supervised classification models for the classification of OpenAI's ChatGPT-generated textual content. Classic supervised machine learning models including Multinomial Naive Bayes, Random Forest, Support Vector Machines (SVM), and K-Nearest Neighbors (KNN) have been trained and tested under various parameters and training regimes. Besides classic supervised machine learning models, a number of deep learning-based models including a baseline Long Short Term Memory (LSTM) model, DistilBERT (Sanh et al., 2019), RoBERTa (Liu et al., 2019), and a custom model have been investigated in this study. All models have been trained using both article-level and sentence-level datasets to identify the impact of document length on the model accuracy. Since these models require different pre-processing, text mining, and feature extraction pipelines, we have organized this section into two subsections including *classic machine learning models* and *deep learning models* as below:

### 5.1 Classic machine learning models

**Figure 4:**
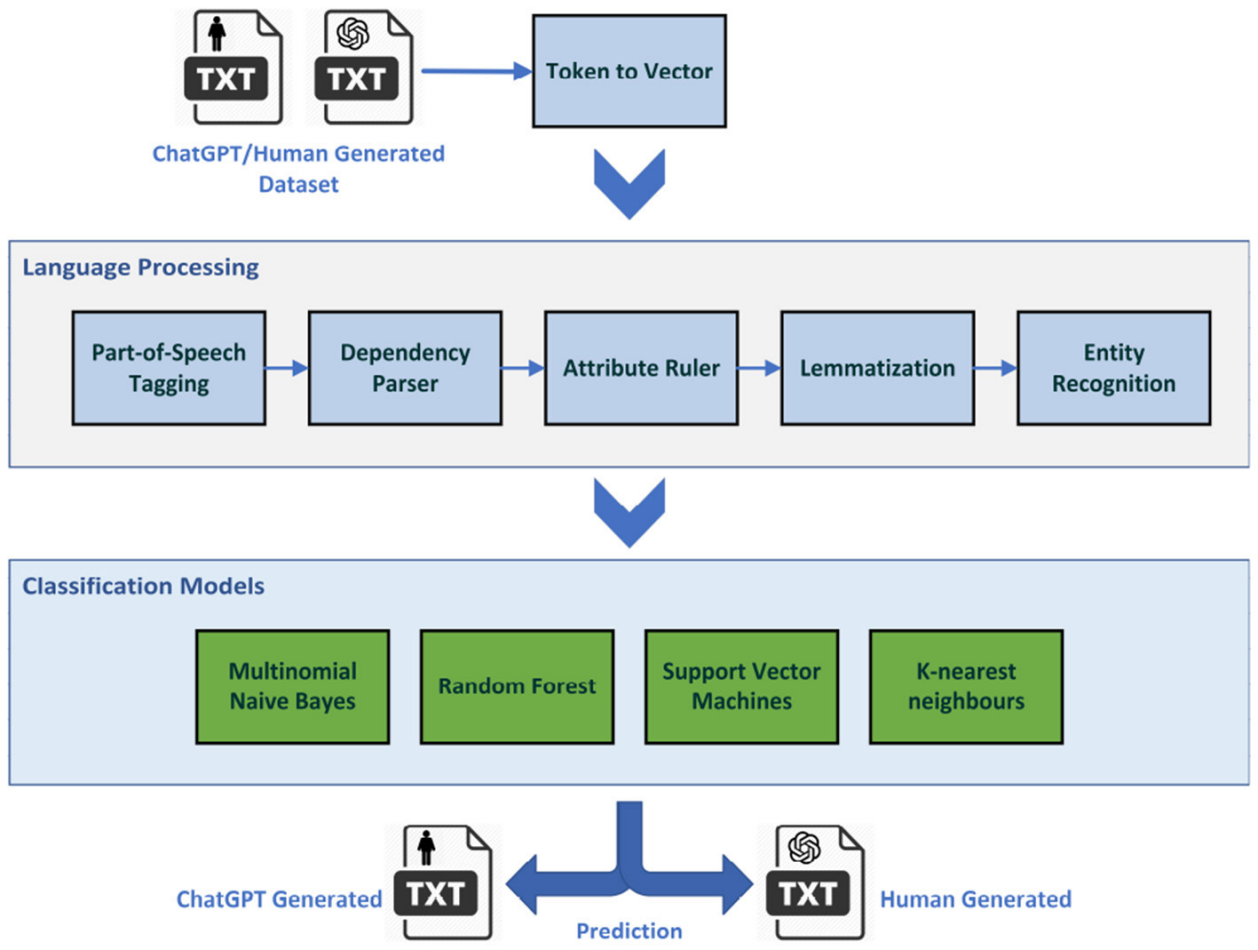
The natural language processing pipeline used in conjunction with classic supervised machine learning models.

Figure 4 shows the NLP pipeline and processes that this study employed in order to transform the raw textual data into a vector of syntactical and sentimental features suitable for the classification process.

In this study, we used the popular Spacy pre-trained English language pipeline which consists of Token to Vector, Part-of-Speech Tagging, Dependency Parser, Attribute Ruler, Lemmatizer, and Entity recognition components. The pipelined pre-trained using *OntoNotes Release 5.0*, a large annotated corpus comprising various genres of text including news, conversational telephone speech, weblogs, Usenet newsgroups, broadcast, and talk shows. The pipeline outputs a feature vector that will be fed into a number of classification algorithms including Multinomial Naive Bayes, Random Forest, Support Vector Machines (SVM), and K-nearest neighbors (KNN) to segregate ChatGPT-generated textual data from human-generated contents. The following section explains each step in more detail.

#### 5.1.1 Natural language processing pipeline

The following section elaborates on the NLP pipeline components, hyperparameters, and procedures that we have utilized to convert raw textual data into a vector of syntactic features suitable for the classification task.

##### 5.1.1.1 Tokenization and vectorization process

Tokenization is the process of breaking up a given text into discrete elements called tokens and Vectorization converts tokenized text data to numerical context-independent word vector representations. We used trainable *HashEmbedCNN.v2* model which consists of a *MultiHashEmbed embedding* layer that uses subword features to construct an embedding layer that embeds lexical attributes using hash embedding. This layer concatenates the results and passes them to subsequent *MaxoutWindowEncoder* layers which encode context using four convolutions with Maxout activation function, layer normalization and residual connections (Vasiliev, 2020). An embedding size of 2,000 was used in this process. This process outputs a vector representation in the form of a tensor that can be used and called by upstream components of the pipeline.

##### 5.1.1.2 Part-of-speech (POS) tagging

POS Tagging includes the assignment of a label (Part-of-Speech) to each word in a text which describes the grammatical function of a word such as nouns, pronouns, verbs, adverbs, etc. The tagger is built on top of the token-to-vector model (HashEmbedCNN.v2), adding a linear layer with Softmax activation to predict Part-of-Speech scores given the token vectors.

##### 5.1.1.3 Syntactic dependency parsing

This analyzes the grammar of the sentence and identifies the connection between words in the sentence, representing them in the form of a tree known as syntactic dependency parsing. The dependency parser is a trainable process which jointly learns sentence segmentation and labeled dependency parsing and can optionally learn to merge tokens that had been over-segmented by the tokenizer. This study employs a variant of the non-monotonic arc-eager transition-based dependency parser proposed by Honnibal and Johnson (2015).

##### 5.1.1.4 Token attribute mappings

A rule-based process that allows setting token attributes for tokens identified by *Matcher patterns* that operates over tokens, similar to regular expressions. The attribute ruler is typically used to handle exceptions for token attributes and helps in a more accurate classification of text.

##### 5.1.1.5 Lemmatization

A non-trainable process that aims for assigning base forms to tokens using rules based on part-of-speech tags, or lookup tables. This study employs the Spacy Lemmatizer component In “lookup” mode based on data available at Spacy (2023).

##### 5.1.1.6 Named entity recognition

This trainable component includes a transition-based named entity recognizer model (Lample et al., 2016) that identifies non-overlapping labeled spans of tokens such as people, organizations, places, etc and assigns the appropriate category.

#### 5.1.2 Classification models

The following section elaborates on various classic supervised machine learning algorithms and their respective hyperparameters that we used in this study.

##### 5.1.2.1 Multinomial Naive Bayes

Multinomial Naive Bayes is a variant of the Naive Bayes algorithm that is particularly used for text classification. It models the frequency of occurrence of features (ex: words/tokens) and makes predictions based on their probabilities, assuming each feature is independent of the others. Multinomial Naive Bayes is suitable for NLP due to its ability to handle high-dimensional, discrete data, such as word counts in text documents. It simplifies complex language problems by assuming feature independence, which makes it efficient for text classification tasks. In our experiments, the smoothing parameter (alpha) is set to 1, and the fit prior parameter is set to True.

##### 5.1.2.2 Random Forest

Random Forest is an ensemble learning model that aggregates multiple decision trees to perform classification. During the training phase of this experiment, a random forest model with 100 trees and a maximum depth of 10 is employed. The algorithm generates 100 decision trees, each built using a random subset of data and features through feature bagging. Each tree includes up to 10 levels of decision nodes to reduce the computational process.

##### 5.1.2.3 Support vector machines (SVM)

SVM is a supervised machine learning algorithm used for both classification and regression applications. It separates classes by finding the hyperplane (decision boundary) that maximizes the margin between them. SVM is popular for NLP due to its effectiveness in handling high-dimensional and its robustness against overfitting. In this study, we used a degree 6 polynomial kernel. The probability parameter (to enable probability estimates) is set to True to remove class size bias and reduce overfitting.

##### 5.1.2.4 K-nearest neighbors (KNN)

KNN is a supervised machine learning algorithm used for classification or regression. KNN predicts a data point's label based on the labels of its “K" closest neighbors in the feature space. In this research the K value is set to 15 (empirically) and the distance metric is set to *euclidean*. KNN is popular for text classification, sentiment analysis, and language modeling due to its capability in capturing semantic similarity by using distance metrics in high-dimensional space.

#### 5.1.3 Training process and parameters

To investigate the impact of document length on classification accuracy, all models have been trained and tested using both article-level and sentence-level datasets. In both cases, the split ratio of 80–20% has been used from training and testing subsets. This results in 875 articles in the training subset and 153 articles in the test subset under the article-level setup. Meanwhile, in the sentence-based setup, the training subset includes 5,875 sentences while the testing subset includes a total of 1,469 sentences. In both scenarios “Human-generated" contents are labeled as 0 while “Chat-GPT-generated" contents are labeled as 1. All subsets were seeded and randomly shuffled for uniformity. Subsets were pickled to maintain consistency and repeatability across experiments. In both article-level and sentence-based setups, the language model pipeline outputs a normalized vector of 300 dimensions with 514k unique vectors and 514k corresponding keys which is more than adequate for the complexity and permutation of data in this study. The feature vector will be fed into aforementioned classifiers including Multinomial Naive Bayes, Random Forest, Support Vector Machines (SVM), and K-nearest neighbors (KNN) to segregate ChatGPT-generated textual data from human-generated contents. Experiment results will be discussed in the *Results and Discussion* section.

### 5.2 Deep learning models

In addition to classic machine learning models, this study investigates several state-of-the-art deep learning model performances for the classification of ChatGPT-generated textual content from human-generated content. These models include a baseline Long Short Term Memory (LSTM) model, DistilBERT (Sanh et al., 2019), RoBERTa (Liu et al., 2019), and a custom model based on RoBERTa. Models like RoBERTa and DistilBERT have demonstrated high accuracy in text classification scenarios, particularly for tasks that require distinguishing subtle differences between text types. These models have been shown to outperform traditional techniques in several studies (e.g., Devlin, 2018; Sanh et al., 2019). All models have been trained using both article-level and sentence-level datasets to identify the impact of document length on the model accuracy. These models necessitate the adoption of relatively different NLP processes, hyperparameters, and pipelines, which will be elaborated in the subsequent section.

#### 5.2.1 Preprocessing and vectorization layer

The preprocessing operation converts raw textual data into a vector of syntactic features suitable for training and evaluating deep learning models. It includes two major processes including Text Vectorization and padding. Text Vectorization is a pre-processing layer that simplifies the task of converting raw text data into numerical representations that can be used as input for deep learning models. It takes the input textual data and performs a series of operations, such as tokenization, standardization including lowercasing, punctuation removal, and vocabulary creation. The Text Vectorization layer then transforms the preprocessed text data into a sequence of integer indices or dense vectors. It provides a convenient and efficient way to preprocess text data and prepare it for further processing in neural networks, however, it doesn't take into account more intricate language elements like morphological variations or subword segmentation. Apart from DistilBERT model, we used TensorFlow's *TextVectorization* layer in combination with *int* output mode parameter which assigns a special integer ID to each token. The *output_sequence_length* parameter sets the length of the output sequences to 200 words which is aligned with the average length of articles in our dataset. If texts are shorter than this length, they get padded with zeros; otherwise, they get truncated. This ensures all sequences have uniform lengths for efficient and unbiased model training. The maximum vocabulary length is set to 20,000 words empirically.

The DistilBERT model benefits from a custom tokenizer that makes use of the WordPiece algorithm. This technique breaks down words into subwords, enabling the model to handle a wide range of vocabularies, including rare and out-of-vocabulary words. Also, it provides better control over morphological variations, enhancing the model's overall efficiency and performance. In this study, we used Keras' *DistilBertTokenizer* and *DistilBertPreprocessor* for DistilBERT deep model. We employed *distil_bert_base_en_uncased* tokenizer preset, which is optimized within a 6-layer DistilBERT model with 66 million parameters where all input is lowercased. This model is pre-trained on English Wikipedia and BooksCorpus using BERT as the teacher model. Besides integer token IDs, the output sequence also includes a list of special tokens including CLS which denotes the start of the sequence token, SEP which represents the end of the sequence token, and PAD which indicates padding tokens, meant to equalize the sequence length across the input data.

#### 5.2.2 Deep models

The following section elaborates on the model architecture and hyperparameters of the employed deep learning models in this study:

##### 5.2.2.1 Bidirectional LSTM based Model

**Figure 5:**
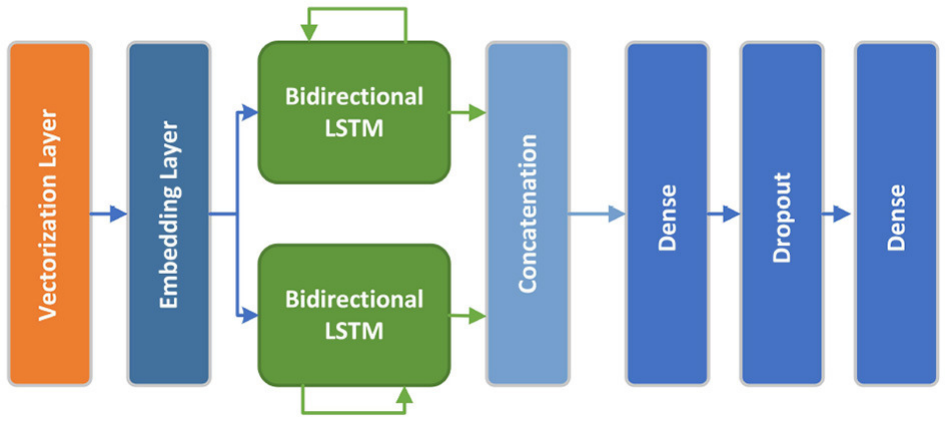
Bidirectional LSTM model architecture.

The bidirectional LSTM model consists of an embedding layer which receives the preprocessed, vectorized and padded sequenced from the *TextVectorization* preprocessing layer. This layer outputs a 128-dimension vector. The embedding layer is followed by two Bidirectional LSTM layers with 128 and 64 dimensions respectively. Next, the model includes a 128-dimensional dense layer with relu activation function, followed by a 20% Dropout layer and a final one-dimensional dense layer with a sigmoid activation function. The model has been paired with *BinaryCrossentropy* loss function and Adam optimizer. Adaptive learning rate and early stopping regularization mechanisms have been arranged to optimize neural network training, enhancing model performance and preventing overfitting. Figure 5 shows the conceptual Bidirectional LSTM model architecture.

##### 5.2.2.2 DistilBERT

**Figure 6:**
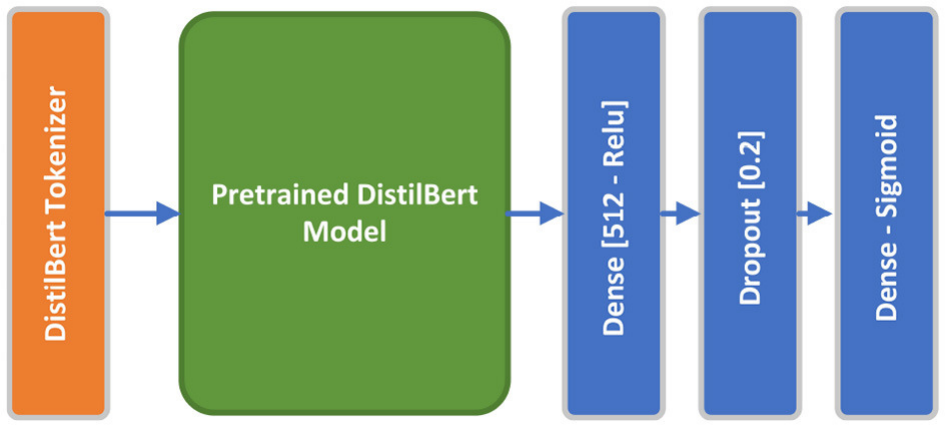
DistilBERT model architecture.

DistilBERT, a condensed, speedy, cost-efficient, and lightweight Transformer model, has been developed by distilling the BERT base model. It features 40% fewer parameters than the bert-base-uncased, allowing it to execute tasks 60% more rapidly, while still retaining above 95% of BERT's effectiveness as evaluated on the GLUE language comprehension benchmark (Sanh et al., 2019). We employed pretrained *distil_bert_base_en_uncased* tokenizer preset, which is optimized within a 6-layer DistilBERT model with 66 million parameters where all input is lowercased, paired with *distilbert-base-uncased* pretrained model. The DistilBERT model which outputs a vector of 768 dimensions, is followed by a 512-dimensional dense layer with relu activation function, a 20% Dropout layer, and a final one-dimensional dense layer with a sigmoid activation function. The pretrained DistilBERT along with the top layers will be finetuned using the proposed dataset during the training process. Figure 6 shows the conceptual DistilBERT model architecture. The model has been paired with *sparse_categorical_crossentropy* loss function and Adam optimizer. Adaptive learning rate and early stopping regularization mechanisms have been arranged to optimize model performance and prevent overfitting. Hyperparameters are fined-tuned empirically to maximize model accuracy.

##### 5.2.2.3 RoBERTa

**Figure 7:**
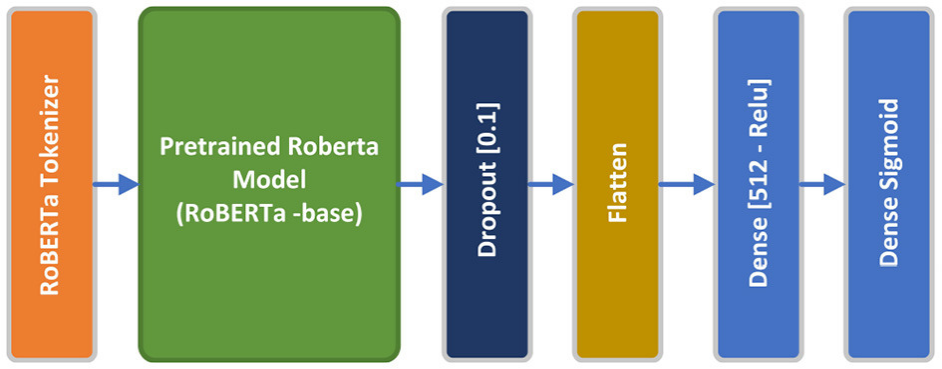
RoBERTa model architecture.

RoBERTa, an evolution of BERT, tweaks key hyperparameters, discards the next-sentence pretraining and employs larger mini-batches and learning rates for enhanced training efficacy. RoBERTa includes minor tweaks to embeddings and pretrained models setup. It employs a byte-level BPE tokenizer, similar to GPT-2, and a bespoke pretraining method. RoBERTa doesn't require *token_type_ids*, instead, segments are separated by a token, such as *tokenizer.sep_token* or < */s>*. The model enhances pretraining by applying dynamic masking, permitting token masking to vary across epochs, unlike BERT. Furthermore, it trains with larger batches and uses BPE with bytes, accommodating Unicode characters. For this experiment, we employed pretrained *roberta_base* tokenizer preset, along with *roberta_base_en* pretrained RoBERTa backbone, which is a 12 encoders layer, case-sensitive model, consisting of 110 million parameters trained on English Wikipedia, BooksCorpus, CommonCraw, and OpenWebText. The RoBERTa model outputs a vector of 768 dimensions. A 10% Dropout layer, Flatten layer, a 512-dimensional dense layer with relu activation function, and a final one-dimensional dense layer with a sigmoid activation function comprise the top layers of the RoBERTa model. The pretrained RoBERTa along with the top layers will be finetuned using the proposed dataset during the training process. Similar to the DistilBERT model, RoBERTa has been paired with *sparse_categorical_crossentropy* loss function and Adam optimizer. Adaptive learning rate and early stopping regularization mechanisms have been arranged to optimize model performance and prevent overfitting. Hyperparameters are fined-tuned empirically to maximize model accuracy. Figure 7 shows the conceptual RoBERTa model architecture.

##### 5.2.2.4 Custom Deep Model

The proposed Custom Deep Model works similarly to the RoBERTa however instead of 12 encoders layers, it benefits from four encoders layers only. On top of that, it uses parameter-sharing across layers and a factorized embedding parameterization to further reduce the model size. These changes reduce the model size down to only 4 million parameters, making it considerably faster than the RoBERTa with over 110 million parameters. The proposed custom model uses the same pretrained BertTokenizer. This model has been trained using English Wikipedia and BooksCorpus and then finetuned using the proposed dataset. The top layer architecture in this model is similar to the one in RoBERTa model. Similarly, the proposed model has been paired using *sparse_categorical_crossentropy* loss function and Adam optimizer with adaptive learning rate and early stopping regularization mechanisms to optimize model performance and prevent overfitting.

## 6 Results and discussion

### 6.1 Classic machine learning models

**Figure 8:**
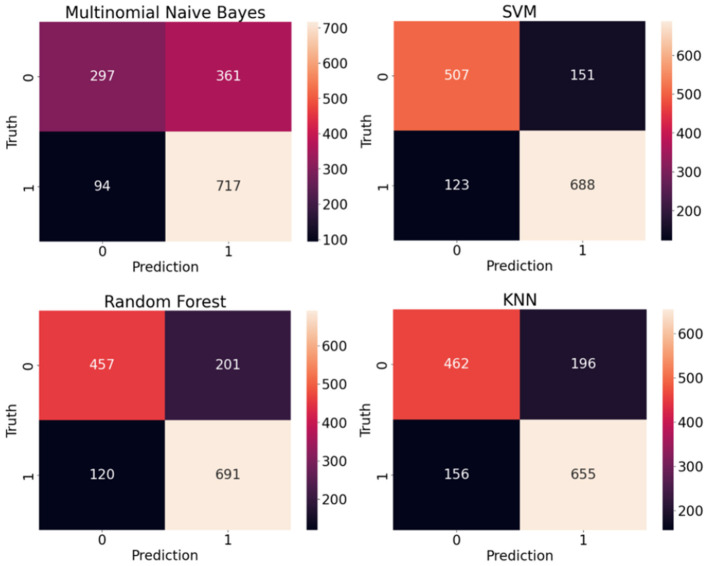
Confusion matrices of classic machine learning models using sentence-level dataset.

**Figure 9:**
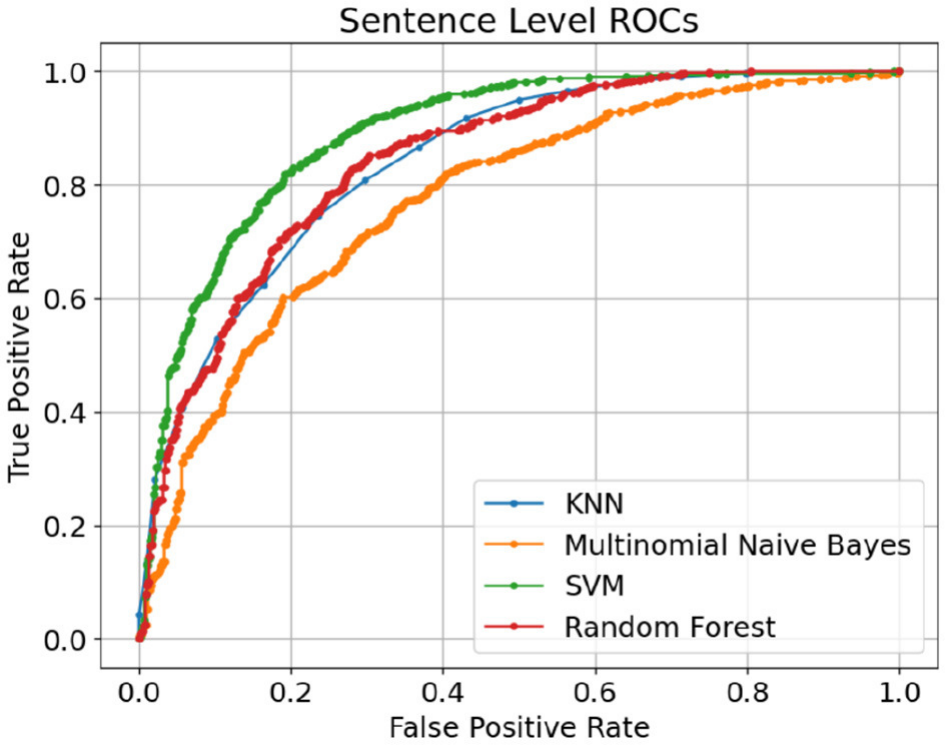
ROC curves of classic machine learning models using sentence-level dataset.

Both classic and deep learning based models are trained and tested on sentence-level and article-level datasets to identify the impact of document length on the model accuracy. The experimental setup and hyperparameters under each experiment have been explained in the previous section. Table 1 shows the performance of classic supervised machine learning models using sentence-level datasets. Support Vector Machine (SVM) supervised algorithm with F1-score and Accuracy of 0.833 and 0.813 respectively outperformed other classic classification algorithms in this study. With a considerable margin, Random forest with F1-score and Accuracy of 0.811 and 0.781 respectively was the second-best performer among classic classification algorithms in this study. As anticipated, Multinomial Naive Bayes with F1-score and Accuracy of 0.759 and 0.690 was the worst performer among classic classification algorithms in this experiment. Given the average length of sentences in our sentence-level datasets is less than 20 words, it is extremely challenging for any supervised classification algorithm to segregate human-generated from AI-generated (ChatGPT) contents due to the lack or even absence of discriminative features in shorter data points. Figure 8 illustrates confusion matrices of classic machine learning models using sentence-level dataset. Although the overall result is not promising, SVM shows a noticeable edge over other classification algorithms in this experiment. Figure 9 displays ROC (Receiver Operating Characteristic) curves of classic classification models using the sentence-level dataset which implies the trade-off between the sensitivity and specificity of the models that we tested in this experiment.

**Table 1:** Performance of classic supervised machine learning models using sentence-level dataset.

	**Precision**	**Recall**	**F1-score**	**Accuracy**
Multinomial Naive Bayes	0.665	0.884	0.759	0.690
SVM	0.820	0.848	0.833	0.813
Random Forest	0.774	0.852	0.811	0.781
KNN	0.769	0.807	0.788	0.760

**Figure 10:**
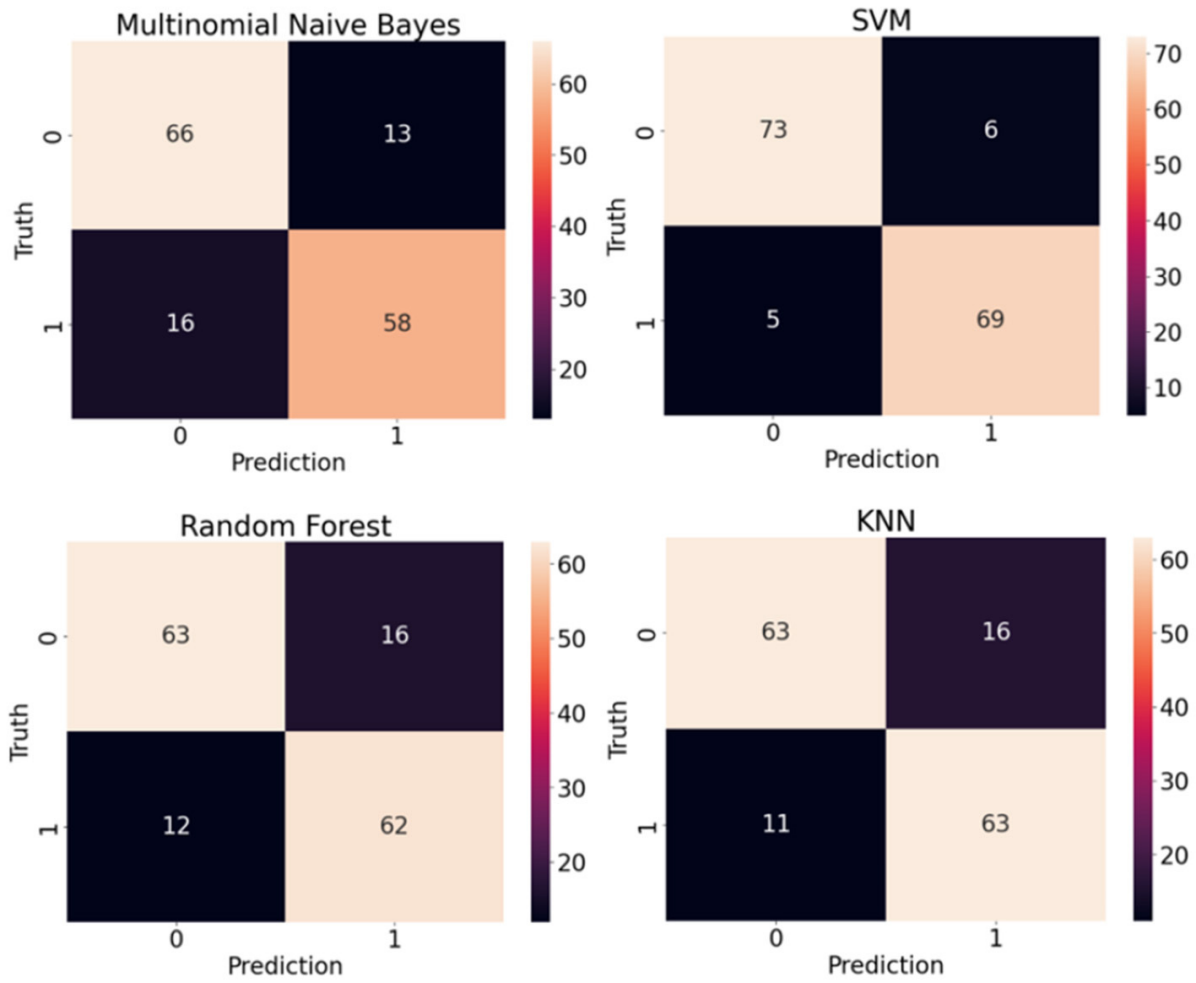
Confusion matrices of classic machine learning models using article-level dataset.

**Figure 11:**
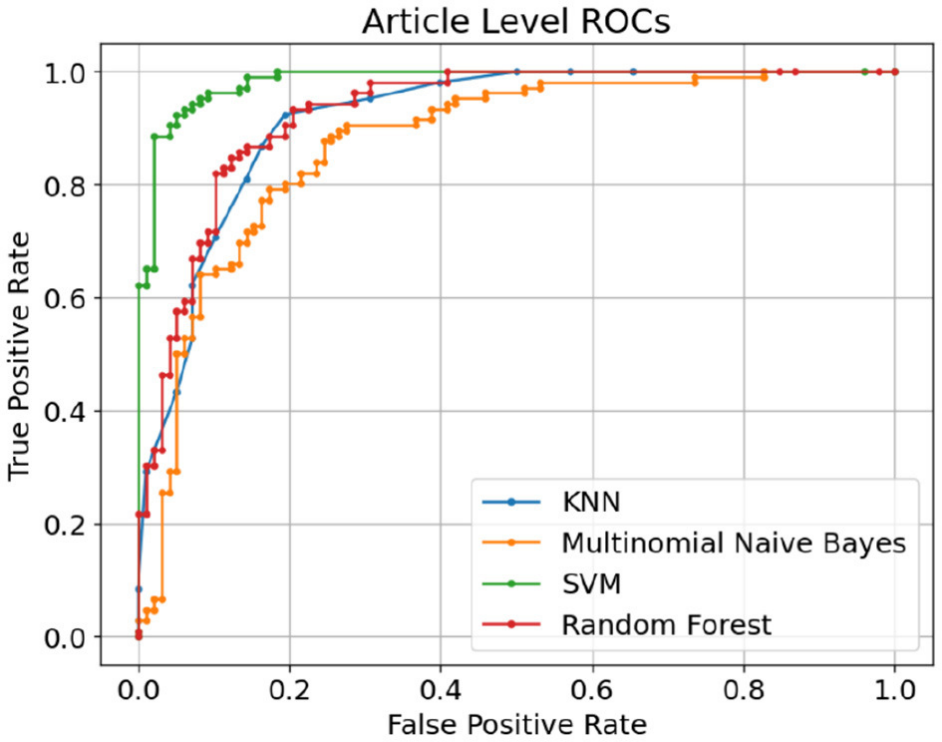
ROC curves of classic machine learning models using article-level dataset.

Table 2 shows the performance of classic supervised machine learning models using article-level dataset. A noticeable boost across all classification metrics is evident when classifying the article-level dataset. The article-level dataset provides substantially larger data points, (approximately 180 words) allowing for the emergence of more discriminative features within these data points which enhances the accuracy of the classification. Similar to sentence-level experiments, SVM with F1-score and Accuracy of 0.926 and 0.928 respectively outperformed other classic classification algorithms in this study. This time around, KNN with F1-score and Accuracy of 0.823 was the second-best performer in this experiment. Once again, Multinomial Naive Bayes with F1-score and Accuracy of 0.80 and 0.810 was the worst performer among classic classification algorithms in this experiment. Figure 10 illustrates confusion matrices of classic machine learning models using article-level dataset. As can be seen, the overall results are significantly better than the experiments over the sentence-level dataset and SVM shows a noticeable edge over other classification algorithms. Figure 11 displays ROC curves of classic classification models using the article-level dataset. Once again, a significant improvement across all performance metrics is evident.

**Table 2:** Performance of classic supervised machine learning models using article-level dataset.

	**Precision**	**Recall**	**F1-score**	**Accuracy**
Multinomial Naive Bayes	0.816	0.783	0.8	0.810
SVM	0.92	0.932	0.926	0.928
Random Forest	0.794	0.837	0.815	0.816
KNN	0.797	0.851	0.823	0.823

Although classic machine learning models, specifically SVM, performed reasonably well with larger, article-level data points, their performance significantly declined when it came to shorter, sentence-level datasets, suggesting a significant potential for improvement. The following section will detail how state-of-the-art deep learning-based models can surpass classic machine learning model's performance in the classification of AI-generated (ChatGPT) content.

### 6.2 Deep learning models

**Figure 12:**
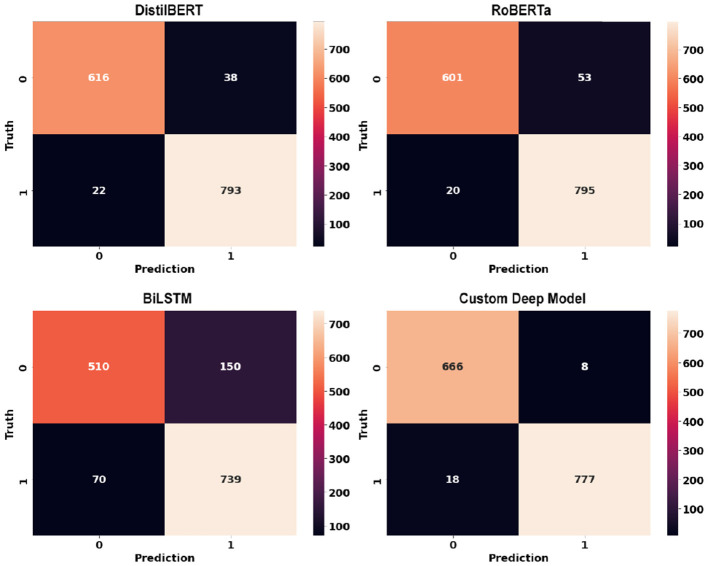
Confusion matrices of deep learning models using sentence-level dataset.

**Figure 13:**
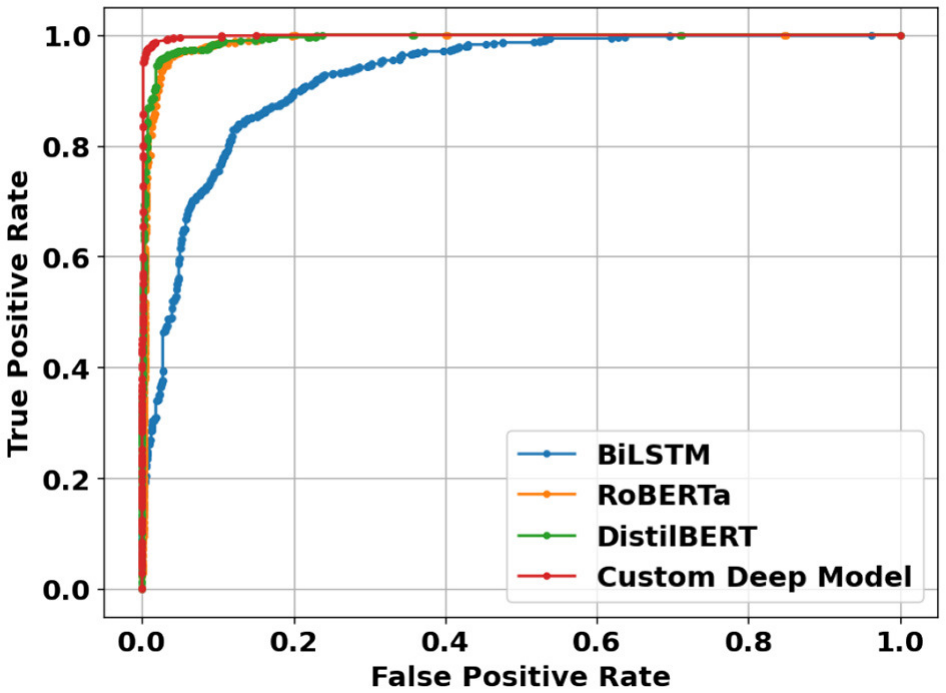
ROC curves of deep learning models using sentence-level dataset.

Table 3 shows the performance of deep learning models using sentence-level dataset. There is an immediate, noticeable boost across all classification metrics when compared to the sentence-level results obtained using classic machine learning models. This demonstrates the superiority of pretrained deep learning models over traditional machine learning models. The proposed RoBERTa based custom deep model, outperformed other deep learning models in sentence-level experiments, achieving an F1 score of 0.981 and an accuracy of 0.983, respectively. With some margin, DistilBERT with F1-score and Accuracy of 0.962 and 0.960 respectively was the second-best performer in this experiment. The base RoBERTa model, however, lagged marginally behind DistilBERT, achieving an F1 score of 0.954 and an accuracy of 0.957. Bidirectional LSTM with F1-score and Accuracy of 0.824 and 0.852 was the worst performer among deep learning models in sentence-level experiments. Overall, deep learning models performed exceptionally well considering the extremely challenging scenario caused by smaller data points (less than 20 words) in sentence-level experiments. Figure 12 illustrates confusion matrices of deep learning models using sentence-level dataset. As can be seen, the overall results are significantly better than similar experiments using classic machine learning models. Figure 13 displays ROC curves of deep learning models using the sentence-level dataset. Once again, a significant improvement across all performance metrics is evident.

**Table 3:** Performance of deep learning models using sentence-level dataset.

	**Precision**	**Recall**	**F1-score**	**Accuracy**
Bidirectional LSTM	0.829	0.832	0.824	0.852
RoBERTa	0.946	0.959	0.954	0.957
Custom deep model	0.977	0.971	0.981	0.983
DistilBERT	0.955	0.961	0.962	0.960

**Figure 14:**
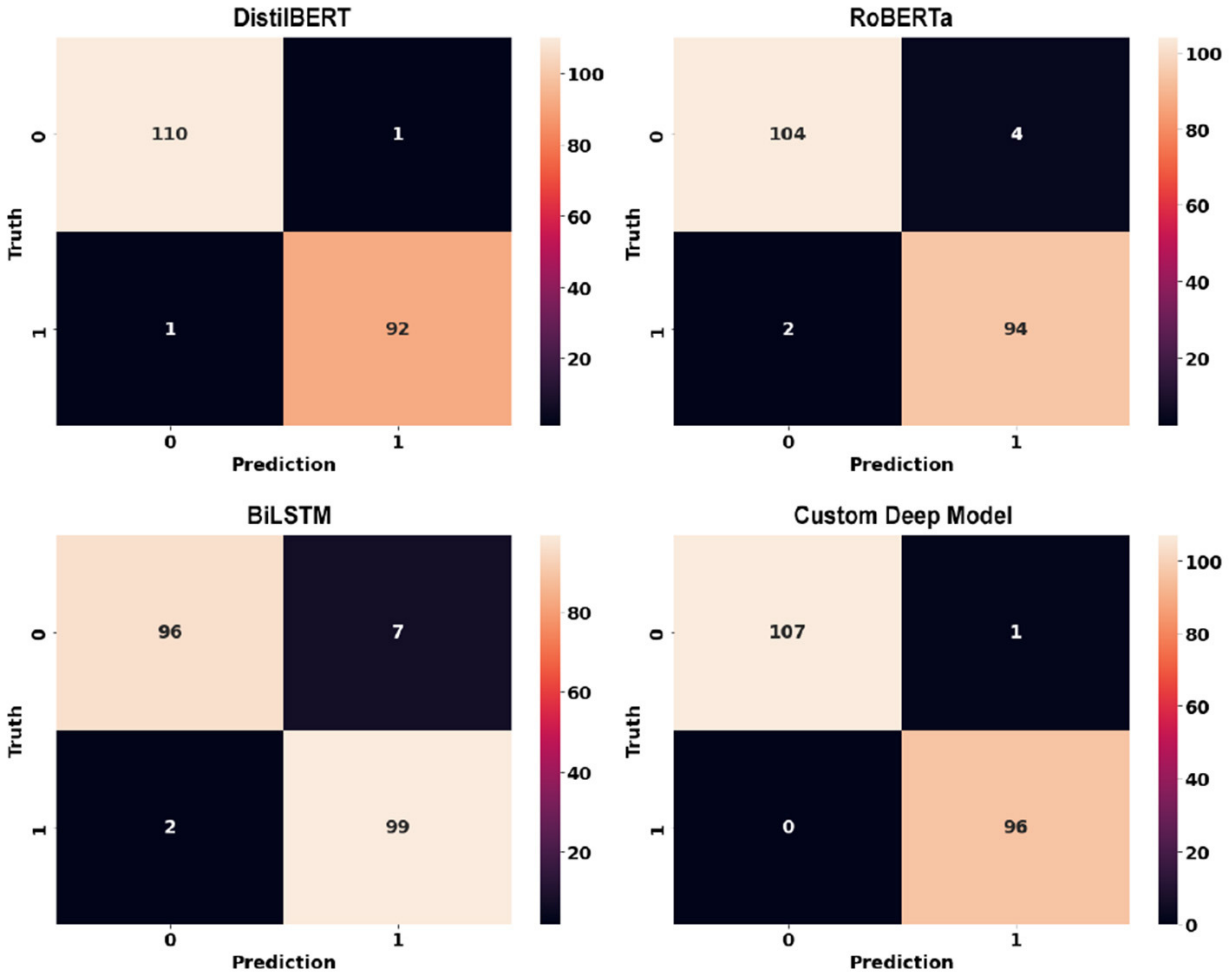
Confusion matrices of deep learning models using article-level dataset.

**Figure 15:**
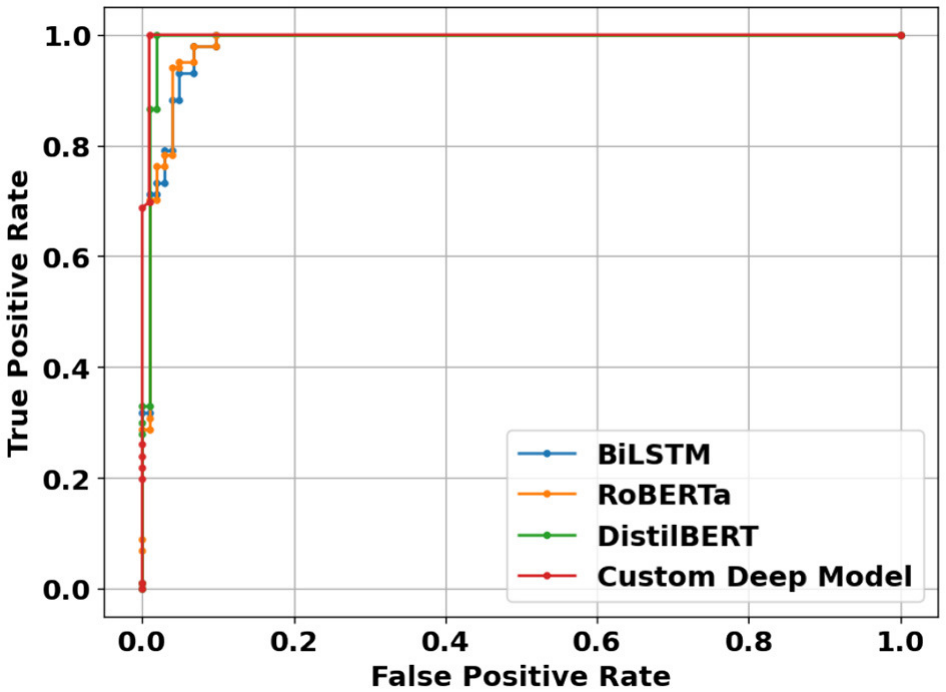
ROC curves of deep learning models using article-level dataset.

Deep learning models can show their true potential When it comes to larger data points in article-level dataset. The article-level dataset offers significantly larger data points (around 180 words each) which allows the emergence of discriminative features within these data points. Table 4 shows the performance of deep learning models using article-level dataset. Once again, there is a noticeable improvement across all classification metrics when compared to similar experiments using classic machine learning models. Similar to sentence-level experiments, The proposed RoBERTa based custom deep model with F1-score and Accuracy of 0.992 and 0.991 respectively outperformed other deep models in this experiment. DistilBERT with F1-score and Accuracy of 0.988 was the second-best performer in this experiment. Similar to sentence-level experiments, Bidirectional LSTM with F1-score and Accuracy of 0.952 and 0.957 showed the least desirable performance among deep learning models in this experiment. Figure 14 illustrates confusion matrices of deep learning models using article-level dataset. As can be seen, deep learning models can perform exceptionally well using the article-level dataset. The proposed RoBERTa based custom deep model misclassified only a single data point in our test set. Figure 15 displays ROC curves of deep learning models using the article-level dataset. Once again, exceptional performance across all metrics is evident.

**Table 4:** Performance of deep learning models using article-level dataset.

	**Precision**	**Recall**	**F1-score**	**Accuracy**
Bidirectional LSTM	0.944	0.951	0.952	0.957
RoBERTa	0.978	0.981	0.981	0.980
Custom deep model	0.989	0.991	0.992	0.991
DistilBERT	0.986	0.988	0.988	0.988

**Figure 16:**
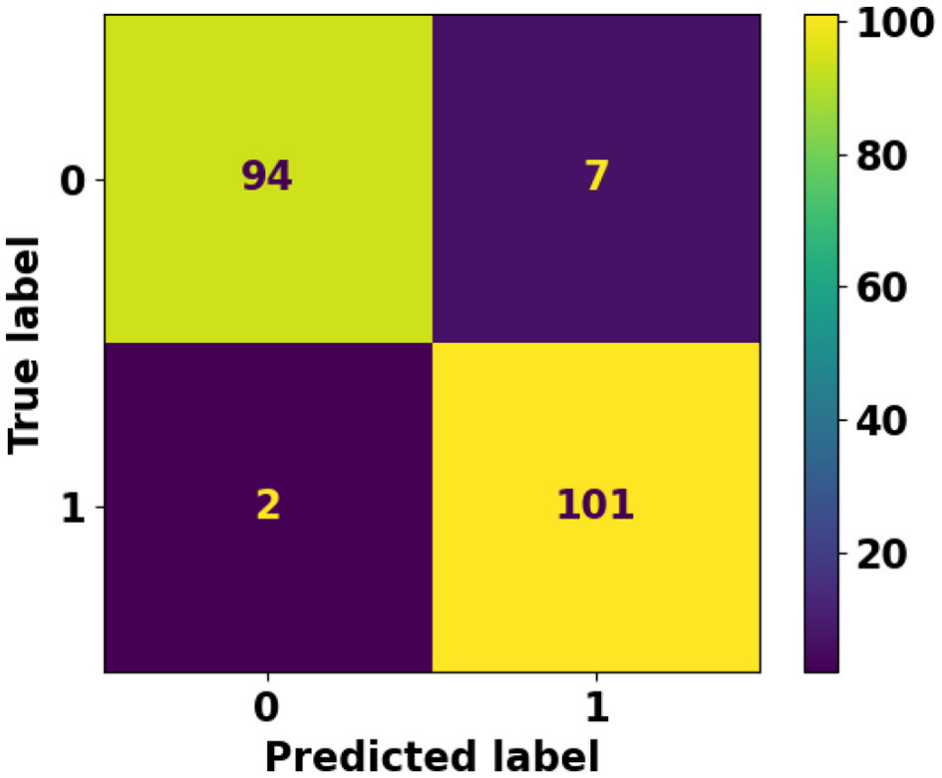
Confusion matrix of Turnitin AI writing detection tool using article-level dataset.

The experiment demonstrates that both classic and deep learning models perform better when working with larger, article-level datasets as opposed to sentence-level datasets. However, the performance of deep learning models significantly outshines that of classic machine learning models in both contexts. For classic machine learning models, the Support Vector Machine (SVM) algorithm showed the best performance in both dataset types, yet struggled with shorter sentence-level data due to a lack of discriminative features. In contrast, deep learning models exhibited superior performance. Particularly, a custom model based on RoBERTa stood out, achieving the highest classification accuracy in both sentence and article-level datasets. This testifies deep learning models' ability in capturing complex patterns in data, making them a preferable choice for the classification of AI-generated (ChatGPT) content. Besides the aforementioned experiments, we attempted to compare our best model performance with the Turnitin AI writing detection feature. Turnitin, has been a leading solution for plagiarism detection, with over 98% of top universities in the UK using this tool. It scans academic work for plagiarism by comparing the work to a large database of student work, publications, and materials on the internet. Turnitin's ability to detect AI-generated content has made it a relevant benchmark in evaluating text classification models for academic integrity, and it serves as a comparison in our study due to its established effectiveness in the academic domain. Figure 16 shows the confusion matrix of Turnitin using the article-level dataset. Unlike the proposed method, Turnitin is unable to investigate articles shorter than 300 words. This limitation significantly cripples its usefulness in many scenarios. Due to this limitation, we are unable to investigate Turnitin AI tool performance using our sentence-level dataset which features shorter data points. Figure 16 also shows Turnitin AI tool achieved a reasonably well False Negative (two misclassified instances only). However, its performance drops when it comes to False Positive (seven misclassified instances). Table 5 shows Turnitin AI tool performance metrics and compares it against the proposed RoBERTa-based custom deep model.

**Table 5:** Performance comparison between the Turnitin AI detection feature and our best model (RoBERTa-based custom deep learning model) using the article-level dataset.

	**Precision**	**Recall**	**F1-score**	**Accuracy**
Turnitin AI tool	0.948	0.950	0.957	0.955
Proposed RoBERTa-based custom model	0.989	0.991	0.992	0.991

As part of our analysis, we examined misclassified samples to identify patterns that may contribute to classification challenges. Since the human-generated texts in our dataset originate from formally published literature, they generally maintain high linguistic quality, minimizing grammatical or spelling errors. Therefore, we do not believe these issues provide any meaningful clues for identifying human-generated content. Our word frequency and similarity analysis revealed subtle differences between AI-generated and human-written text, indicating that certain lexical and stylistic features impact classification, as further supported by our examination of the average cosine and Jaccard similarity indices between misclassified and correctly classified texts for both AI-generated and human-generated content. The results indicate that misclassified texts tend to exhibit higher overall similarity between AI and human-generated classes. However, we cannot confirm whether these similarities are the primary cause of misclassification by our deep learning models. Table 6 summarize the similarity analysis between AI generated and human generated contents for both misclassified and correctly classified samples.

**Table 6:** Average Jaccard and Cosine similarity for correctly and incorrectly classified instances of human-generated and AI-generated text. Incorrectly classified instances exhibit noticeably higher similarity.

	**Correctly classified instances**	**incorrectly classified instances**
Average cosine similarity	0.167	0.197
Average Jaccard similarity	0.023	0.031

### 6.3 Limitations in dynamic word generation of AI models

Limitations in Dynamic Word Generation of AI Models: While AI models such as RoBERTa and DistilBERT demonstrate exceptional performance in detecting AI-generated content, there remain inherent limitations in their ability to capture the nuanced differences between dynamic word generation by AI and human authors. AI-generated content, particularly from large language models like ChatGPT, often lacks the depth of contextual understanding and creativity that human writers bring to their work. This results in subtle yet significant disparities in narrative coherence, idiomatic expressions, and contextual relevance. Moreover, AI models may struggle with generating highly dynamic or context-specific content, such as abstract reasoning or emotionally charged writing, which often rely on personal experience, culture, or intricate domain knowledge. These limitations can affect the models' ability to fully differentiate between AI and human content, especially in tasks that require more sophisticated and adaptable language use. Future iterations of detection models will need to consider these limitations to improve robustness in differentiating content, particularly in domains where creativity and personal voice are critical.

### 6.4 Limitations in model generalizability

While our dataset includes both AI-generated and human-generated content, it is primarily focused on specific domains, particularly computer science and networks. This limitation may affect the generalizability of our findings to other fields or types of content, as language, writing styles, and contextual nuances can vary significantly across different domains. Consequently, the applicability of our proposed model may be restricted when applied to areas outside of computer science, which could limit its effectiveness in detecting and classifying AI-generated content in diverse contexts. Acknowledging this constraint is crucial, as it highlights the need for further research to validate the model's performance across a broader range of disciplines and content types. Future studies should consider expanding the dataset to include a more diverse array of domains to enhance the model's generalizability and robustness in various applications.

Also, the reliance on a specific set of AI-generated content, namely OpenAI's ChatGPT, presents a significant limitation in this study. While there are several other AI models for generating textual content, such as Google Gemini and Facebook LLaMA, our research focused solely on ChatGPT due to its popularity and widespread use. This narrow focus raises questions about the adaptability of our model to outputs generated by other AI systems, as different models may produce varied outputs due to differences in their architectures. Consequently, the generated text may vary significantly in terms of word frequency and intrinsic statistical features, which could affect the model's performance in detecting and classifying content from these alternative generative models. This limitation is inherently embedded in our dataset and represents a constraint of our research, emphasizing the need for further exploration of the model's applicability across various AI-generated content types.

Another worth-mentioning challenge in detecting AI-generated text is the continuous evolution of generative models, which refine their outputs to better mimic human writing. As a result, the effectiveness of detection models may diminish over time if not periodically updated. Our proposed model demonstrates strong performance based on the data available at the time of experimentation; however, we acknowledge that ongoing retraining with newly generated text is essential to maintain accuracy. This limitation is inherent to all classification models in this domain, underscoring the need for adaptive strategies and regular updates to ensure long-term robustness.

### 6.5 Limitations in detecting shorter text entries

The study identifies a notable performance gap in detecting shorter text entries, particularly within the sentence-level dataset. While our models demonstrate strong efficacy with longer, article-level data, their performance diminishes significantly when applied to shorter samples. This reduction in performance is primarily attributed to the lack of discriminative statistical features in shorter data points, which makes it challenging for the models to accurately classify content. This limitation is particularly relevant in real-world scenarios, where shorter text entries are common. Notably, this challenge is not exclusive to our proposed models; it is also observed in commercial solutions like Turnitin, which requires a minimum length of 300 words for its AI detection features to function effectively. This emphasizes the inherent difficulty of accurately detecting AI-generated content in brief texts, a factor that urges further investigation and highlights the need for developing models that can better address shorter entries.

## 7 Conclusion

The rapid advancement of AI, particularly artificial neural networks, has created revolutionary breakthroughs and applications, including text-generating tools or chatbots. However, this powerful technology also brings with it potential misuse and societal implications, such as violation of privacy, misinformation, and challenges in academia. Hence, this research aimed to investigate the performance of various machine learning and deep learning models in detecting and classifying AI-generated content, specifically focusing on the textual content generated by OpenAI's ChatGPT. A fairly large dataset of both human and ChatGPT-generated content was compiled and utilized to train and evaluate several machine learning and deep learning models under different training regimes. Among the models evaluated, the RoBERTa-based custom deep learning model significantly outperformed other models in classifying content at both the sentence and article levels. The main contributions of this study include the creation and compilation of a large, diverse dataset consisting of both human and AI-generated (specifically, ChatGPT-generated) textual content in the field of computer science and networks. This dataset is a crucial resource for training and testing machine learning and deep learning models aimed at distinguishing AI-generated content. This dataset is made publicly available for the research community. Another key contribution of this study is the comprehensive evaluation and comparison of various classic machine learning and deep learning models on the task of classifying AI-generated vs. human-generated content. The study spans different types of models, from Support Vector Machines and Random Forest to advanced deep learning models like RoBERTa, DistilBERT, and BiLSTM. Our work establishes a robust baseline for the detection and classification of AI-generated textual content, contributing a crucial step toward mitigating the potential misuse of AI-powered text generation tools. Furthermore, the dataset compiled for this research has been made publicly available, serving as a valuable resource for future research in this field. In conclusion, the ability to generate realistic and contextually appropriate text raises concerns about the spread of misinformation, manipulation, and malicious activities, where individuals may exploit these technologies for deceptive purposes. Moreover, detection tools and algorithms themselves are not immune to misuse; they can be subjected to adversarial attacks, where malicious actors manipulate inputs to evade detection, undermining the very systems intended to ensure integrity. This raises critical ethical issues, including the potential for biased or inaccurate outcomes, unjust penalization of legitimate content, and erosion of trust in both AI-generated and human-authored texts. To address these challenges, it is essential for researchers, educators, and policymakers to develop comprehensive guidelines that promote responsible usage of AI technologies, fostering a balanced approach that safeguards against misuse while harnessing the benefits of innovation. The primary goal of our work was to demonstrate the feasibility of accurately detecting AI-generated content, establishing a foundation for future measures to address the potential misuse of AI-powered text generation tools. While our results do not directly prevent misuse, the development of reliable detection tools is a crucial step toward managing such risks. A high-performing detection model enables educators, researchers, and online platforms to identify and differentiate AI-generated content, helping to safeguard academic integrity, reduce misinformation, and protect against privacy violations. This capability is essential for addressing the societal challenges posed by AI-driven text generation.

While current study demonstrates effective detection of AI-generated text, our future work will focus on expanding the dataset to include a broader range of disciplines, such as medicine, healthcare, news, and humanities, to enhance the generalizability of detection models. Additionally, we aim to develop advanced classification techniques that can distinguish AI-generated text using multiple leading generative models beyond OpenAI's ChatGPT. Another key direction involves analyzing which state-of-the-art generative AI models produce content that is most human-like, presenting greater challenges for detection. To support further research in this field, we plan to publicly share the dataset used in our future studies, fostering collaboration and innovation in AI-generated text detection.

## Data Availability

The datasets presented in this study can be found in online repositories. The names of the repository/repositories and accession number(s) can be found in the article/supplementary material.
